# CdgC, a Cyclic-di-GMP Diguanylate Cyclase of *Azospirillum baldaniorum* Is Involved in Internalization to Wheat Roots

**DOI:** 10.3389/fpls.2021.748393

**Published:** 2021-10-20

**Authors:** Daniel Sierra Cacho, David S. Zamorano Sánchez, Maria Luisa Xiqui-Vázquez, Víctor Iván Viruega Góngora, Alberto Ramírez-Mata, Beatriz E. Baca

**Affiliations:** ^1^Centro de Investigaciones en Ciencias Microbiológicas, Benemérita Universidad Autónoma de Puebla, Ciudad Universitaria, Puebla, Mexico; ^2^Programa de Biología de Sistemas y Biología Sintética, Centro de Ciencias Genómicas, Universidad Nacional Autónoma de México, Cuernavaca, Mexico

**Keywords:** diguanylate cyclase, extracellular polysaccharides, wheat endophyte colonization, *Azospirillum baldaniorum*, cyclic di-GMP

## Abstract

*Azospirillum baldaniorum* is a plant growth-promoting rhizobacterium (PGPR) capable of fixing nitrogen, the synthesis of several phytohormones including indole-acetic acid, and induction of plant defenses against phytopathogens. To establish a successful and prolonged bacteria-plant interaction, *A. baldaniorum* can form biofilms, bacterial communities embedded in a self-made matrix formed by extracellular polymeric substances which provide favorable conditions for survival. A key modulator of biofilm formation is the second messenger bis-(3′–5′)-cyclic-dimeric-GMP (c-di-GMP), which is synthesized by diguanylate cyclases (DGC) and degraded by specific phosphodiesterases. In this study, we analyzed the contribution of a previously uncharacterized diguanylate cyclase designated CdgC, to biofilm formation and bacterial-plant interaction dynamics. We showed that CdgC is capable of altering c-di-GMP levels in a heterologous host, strongly supporting its function as a DGC. The deletion of *cdgC* resulted in alterations in the three-dimensional structure of biofilms in a nitrogen-source dependent manner. CdgC was required for optimal colonization of wheat roots. Since we also observed that CdgC played an important role in exopolysaccharide production, we propose that this signaling protein activates a physiological response that results in the strong attachment of bacteria to the roots, ultimately contributing to an optimal bacterium-plant interaction. Our results demonstrate that the ubiquitous second messenger c-di-GMP is a key factor in promoting plant colonization by the PGPR *A. baldaniorum* by allowing proficient internalization in wheat roots. Understanding the molecular basis of PGPR-plant interactions will enable the design of better biotechnological strategies of agro-industrial interest.

## Introduction

Plant growth-promoting rhizobacteria (PGPRs) are a group of bacteria that are able to promote growth and increase plant yield through several mechanisms, such as nitrogen fixation, phosphate solubilization, induction of the plant immune response against pathogens, and phytohormone production (García-Fraile et al., 2015; Mhlongo et al., 2018). One of the most widely studied genera and species of this group is *Azospirillum brasilense*, a free-living and nitrogen-fixing bacterium that can colonize important agronomic crops (Compant et al., 2010). It has been established that different *Azospirillum* species can colonize plant roots differently; for instance, *A*. *brasilense* Sp7 is able to adhere to the root surface, forming bacterial aggregates or biofilms (Viruega-Góngora et al., 2020), while *A*. *baldaniorum* (first noted as *Azospirillum brasilense* Sp245; Ferreira et al., 2020) is capable of undergoing internalization and living in the intercellular spaces of plant roots (Schloter and Hartmann, 1998). Plant colonization involves a series of distinct lifestyle choices, including chemotaxis and spatial colonization transitioning to biofilm formation, where cells abandon motility to support a sessile lifestyle (McDougald et al., 2012; Vlamakis et al., 2013).

The second messenger bis-(3′–5′)-cyclic-dimeric-GMP (c-di-GMP), acts to link the perception of diverse environmental signals to alterations in bacterial behavior including motility and biofilm formation. C-di-GMP is synthesized by diguanylate cyclases (DGC) and degraded by specific phosphodiesterases (PDE). DGC enzymes show an active site including a GG(D/E)EF conserved motif, while PDE can be divided in two families, those who possess an EAL motif and those with a HD-GYP motif (Römling et al., 2013, 2017). It has been proposed that the number of DGC/PDE enzymes in microorganisms might be influenced by their ecological niche (Cooley et al., 2016). Soil bacteria such as *Sinorhizobium meliloti* or *Bradyrhizobium japonicum*, have 21 and 51 c-di-GMP metabolizing proteins, respectively, which could imply that a diversity of c-di-GMP signaling-modules might be important for decision-making processes in complex environments such as the soil or the rhizosphere (Gao et al., 2014; Schäper et al., 2016). The *A. baldaniorum* Sp245 genome has 35 genes encoding putative DGC/PDE enzymes, for most of which the physiological role is unknown (Ramírez-Mata et al., 2018). Bioinformatic analysis of the domain architecture of DGCs and PDEs can aid in making an informed prediction of the potential role of these signaling proteins. DGCs and PDEs have frequently been reported to include different accessory domains located at their N-terminal regions, which function as signaling domains capable of sensing environmental conditions, including nutrients, nitric oxide (NO), oxygen (O_2_), light, voltage, redox compounds, and phytohormones (Zähringer et al., 2013). DGCs and PDEs have also been found as part of the prevalent signal transduction family of proteins known as two-component systems, composed of a sensor with a histidine kinase domain (HK) and a response regulator with a receiver domain (REC) typically fused to an effector domain with DNA-binding, protein-binding or enzymatic activity (Galperin, 2010). The REC domain of response regulators is subject of phosphorylation at a conserved aspartate residue which typically affects its activity as an effector. Two of the best characterized REC-containing DGCs are PleD from *Caulobacter crescentus* and WspR from *Pseudomonas aeruginosa.* PleD is involved in morphogenesis and developmental control in *C. crescentus* and its activity as an effector is regulated by the histidine kinase DivJ (Aldridge et al., 2003; Paul et al., 2004, 2008). WspR is an important regulator of the biosynthesis of the exopolysaccharide Pel, which is a key component of *P. aerugino*sa biofilms. The phosphorylation state of WpsR is regulated by a chemotaxis-like signal transduction system that responds to surface-sensing (Hickman et al., 2005; Güvener and Harwood, 2007; Huangyutitham et al., 2013). In *C. crescentus*, the PleD protein occurs in two tandem REC domains linked to the DGC domain, whereas the WspR protein of *P. aeruginosa* includes a REC domain adjacent to its DGC domain.

In this work, we characterized a DGC that is the product of the *cdgC* gene from *A. baldaniorum*, implicated in the process of biofilm formation and plant colonization. CdgC was found to be a functional DGC involved in the production of exopolysaccharides, a role that is likely linked to its requirement for endophytic root colonization by *A. baldaniorum*.

## Materials and Methods

### Bacterial Strains, Culture Media, and Growth Conditions

The bacterial strains used in this work are described in Table 1. *Escherichia coli* strains were grown at 37°C in lysogeny broth (LB) supplemented with 100 μg/mL ampicillin (Ap), 15 μg/mL tetracycline (Tc), 50 μg/mL kanamycin (Km), chloramphenicol (Cm), 10 or 30 μg/mL gentamycin (Gm) when needed. *E. coli* DH5α and *E. coli* S17.1 were used for transformation and conjugation assays, respectively. The *Azospirillum baldaniorum* Sp245 used in this study was kindly provided by Dr. C. Elmerich (Institute Pasteur Paris France). The *Azospirillum* strains were grown at 30°C in K-malate minimal medium [(containing KH_2_PO_4_, 0.87 g; K_2_HPO_4_, 1.67 g; NaCl, 0.2 g; MgSO_4_⋅7H_2_O, 0.01 g; MnSO_4_⋅H_2_O, 0.01 g; FeCl_3_, 0.01 g; Na_2_MoO_4_⋅H_2_O, 0.02 g) L^–1^]; pH 6.8, malic acid, 37.2 mM, and NH_4_Cl 18.6 mM as carbon and nitrogen source, respectively (Ramírez-Mata et al., 2016), Congo red (CR) (Rodriguez Caceres, 1982), LB^∗^ (Lysogeny broth supplemented with 10 mM, CaCl_2_, and 10 mM, MgCl_2_), supplemented with Tc, Km or Gm (15, 50, and 30 μg/mL, respectively), depending on the required, Nitrogen-Free Medium [(NFB, malic acid as carbon source, 4 mL vitamin solution (biotin, 10 mg; pyridoxal-HCl, 20 mg), Fe-EDTA (0.065 g L^–1^), and micronutrient solution (CuSO_4_.5H_2_O, 0.04 g; ZnSO_4_.7H_2_O, 0.12 g; H_3_BO_3_, 1.40 g; Na_2_MoO_4_.2H_2_O, 1.0 g; MnSO_4_. H_2_O, 1.175 g) L^–1^)] (Baldani et al., 1987) or NFB^∗^ modified to achieve a relation C:N = 2 using malic acid at 27.6 mM and supplemented with13.8 mM KNO_3_ as N source (Arruebarrena Di Palma et al., 2013) media.

**TABLE 1:** Bacterial strains used in this study.

**Strain**	**Characteristics**	**References**
*E. coli* DH5α	*F^–^ endA1 glnV44 th-1 recA1 relA1 gyrA96 deoR nupG* ϕ*80dlacZ*Δ*M15* Δ(*lacZYA-argF*)*U169*, *hsdR17*(*r_*K*_^–^ m_*K*_^+^*), λ	Thermo Fisher Scientific
*E. coli* S17.1	*recA thi pro hdsR4* (*r_*K*_^–^ m_*K*_^+^*) (RP4-2T::M-Km::Tn7) Tp^r^ Sm^R^ λpir	Simon et al., 1983
*E. coli* S17.1 pDZ-119 pQE-CdgA	Derivative strain of *E. coli* S17.1 harboring the pLIL-2 vector and the c-di-GMP biosensor pDZ-119	This study
*E. coli* S17.1 pDZ-119 pGEX-CdgC	Derivative strain of *E. coli* S17.1 harboring the pGEX-*cdgC* vector and the c-di-GMP biosensor pDZ-119	This study
*E. coli* S17.1 pDZ-119 pGEX	Derivative strain of *E. coli* S17.1 harboring the pGEX-4T-1 vector and the c-di-GMP biosensor pDZ-119	This study
*E. coli* S17.1 pDZ-119	Derivative strain of *E. coli* S17.1 harboring the c-di-GMP biosensor pDZ-119	This study
*Azospirillum baldaniorum* Sp245	Wild-type strain isolated from wheat roots	Baldani et al., 1987
*A. baldaniorum* 59C	Derivative strain Δ*cdgC* of *A. baldaniorum*	This study
*A. baldaniorum* C21	Derivative strain from *A. baldaniorum* 59C harboring the plasmid pAZBR-*cdgC*, Gm^R^	This study
*A. baldaniorum* C87	Derivative strain from *A. baldaniorum* 59C harboring the empty plasmid pAZBRT7, Gm^R^	This study
*A. baldaniorum* 3A13	Derivative strain from *A. baldaniorum* 59C harboring the plasmid pJB-*cdgC*, Km^R^	This study
*A. baldaniorum* 3D46	Derivative strain from *A. baldaniorum* 59C harboring the empty plasmid pJB3Tc20	This study
*A. baldaniorum* -GFP	Wild-type strain harboring the pMP2444 vector which contains the *eGFP* gene, Gm^R^	This study
*A. baldaniorum* 59C-GFP	Derivative strain from *A. baldaniorum* 59C harboring the pMP2444 vector which contains the *eGFP* gene, Gm^R^	This study
*A. baldaniorum* 2A65	Derivative strain from *A. baldaniorum* 3A13 harboring the pMP2444 vector which contains the *eGFP* gene, Gm^R^	This study
*A. baldaniorum* 2D8	Derivative strain from *A. baldaniorum* 3D46 harboring the pMP2444 vector which contains the *eGFP* gene, Gm^R^	This study
*A. baldaniorum* 2449	Derivative strain from *A. baldaniorum* harboring the pMP2449-5 vector which contains the *mCherry* gene, Gm^R^	Ramirez-Mata et al., 2018
*A. baldaniorum* 2450	Derivative strain from *A. baldaniorum* 59C harboring the pMP2450 vector which contains the *eGFP*, Km^R^	This study

### Bioinformatics Analysis and the CdgC Three-Dimensional Model

The domain architecture of CdgC was analyzed using InterPro (Mitchell et al., 2019) and SMART (Letunic and Bork, 2018) databases. Protein sequence alignment was performed using Clustal Omega (Sievers et al., 2011) and Multiple Align Show (Stothard, 2000) software. WspR (PA3702) (De et al., 2008) from *P. aeruginosa* (PDB accession number 3BRE) and PleD (Wassmann et al., 2007) from *C. vibroides* CB15 (PDB accession number 2V0N) were used as reference sequences in pairwise or multiple sequence alignments. The secondary structure of CdgC was predicted using the Phyre2 (Kelley et al., 2016) software. Three-dimensional structures were modeled employing the I-Tasser server (Yang et al., 2014), and figures were made using the UCSF Chimera program (Pettersen et al., 2004). Logos of active and inhibitory sites were created with WebLogo 3 Create (Crooks et al., 2004).

### Oligonucleotides, Plasmids Constructions, and Generation of Strain Variants

The oligonucleotides and plasmids used are listed in Table 2. The deletion construct pCDR-Δ*cdgC* was generated through the assembly of upstream and downstream flanking regions of 509 nucleotides including the GGDEF domain of *cdgC* in the suicide plasmid pCDR. These sequences were amplified through PCR using primer pairs CdgC13/14 and CdgC17/18. Amplicons were cloned into the vector pGEM T-Easy (Promega) and subcloned into pCDR after digesting with the restriction enzymes (REs) *Eco*RI/*Xba*I and *Not*I/*Xba*I, respectively, and ligating using a T4 DNA ligase (Promega). Gene deletion was achieved through double homologous recombination as previously reported (Ramirez-Mata et al., 2018).

**TABLE 2:** Oligonucleotides and plasmids used in this study.

**Oligonucleotides**	**Sequence**	**References**
CdgC13	5′ GCTCATTGCAGGGCGATGAT 3′	This study
CdgC14	5′ CCATCTAGATGGTTCTGCTTCCCAACACCG 3′	This study
CdgC17	5′ AAGTCTAGAAAGGATTCGGCCTGGGCAAG 3′	This study
CdgC18	5′ TCGGGTGCTGTCCCGTCA 3′	This study
Pr100206F	5′ GGTACCGCTGTCCCCTCTCGCGTT 3′	This study
Pr100206R	5′ GAATTCCTAAGGGGAGGGGACCACGC 3′	This study
*cdgC*-F	5′ TTGAATTCATGCGCGTGCTCATCGCC 3′	This study
*cdgC*-R	5′ TCGTCGACCTAAGGGGAGGGGACCACGC 3′	This study
GmF	5′ TGCCTCGAGGCACCTTGTCGCCTTGCGTA 3′	This study
GmR	5′ TGACTAGTCCTGGCGGCGTTGTGACAATT 3′	This study
**Plasmids**		
pGEM-T Easy	Cloning vector *ori* f1, *lacZa*, Ap^R^	Promega Incorporated
pGEX-4T-1	Expression vector, *tac* promoter, GST tag, Ap^R^	Promega Incorporated
pLIL-2	Expression vector derived from pQE31 containing the *cdgA* gene, Cm^R^, Ap^R^.	Ramírez-Mata et al., 2016
pCDR	Suicide vector. *lacZ*, *traJ*, ColE1, mob^+^, OriT, *sacB*, Tc^R^.	This study
pJB3Tc20	RK2 OriT, Tc^R^, Ap^R^.	Blatny et al., 1997
pJMS-Km	Suicide vector derived from pSUP202, Km^R^.	Ramírez-Mata et al., 2016
pJB3-Km^R^	Plasmid derived from pJB3Tc20, *lac* promoter and Tc^R^ were excised employing *Sal*I enzyme, Km^R^ was cloned into an *Eco*RV site	This study
pBBR1MCS-5	Plasmid derived from pBBR1MCS, *lacZ*-α, T7 promoter, Gm^R^.	Kovach et al., 1994
pAZBR-T7mCh	Suicide vector devired of pJMS-Km, *mCherry* gene, ΩT7 terminator, Km^R^, Tc^R^.	Cruz-Pérez et al., 2021
pAZBR-T7-Gm	Suicide vector derived of pAZBR-T7mCh, ΩT7 terminator, Tc^R^, Gm^R^	This study
pDZ-119	C-di-GMP biosensor, Cm^R^	Martínez-Méndez et al., 2021
pMP2444	Wide host range vector containing the *eGFP* gene expressed under the *lac* promoter, Gm^R^	ClonTech
pMP2449-5	Plasmid derived from pMP2444 with the *eGFP* gene replaced by the *mCherry* gene, Gm^R^	Ramirez-Mata et al., 2018
pMP2450	Plasmid derived from pMP2444, Km^R^	This study
pGEM-Gm^R^	Derivative from pGEM T-Easy containing the gentamicin resistance gene, Ap^R^, Gm^R^	This study
pGEM-FrgA	Plasmid derived from pGEM T-Easy containing an 829 bp fragment corresponding to the upstream sequence of the *cdgC* gene, Ap^R^	This study
pGEM-FrgB	Plasmid derived from pGEM T-Easy containing an 864 bp fragment corresponding to the downstream sequence of the *cdgC* gene, Ap^R^	This study
pGEM-*cdgC*	Plasmid derived from pGEM T-Easy containing the ORF of *cdgC* gene, Ap^R^	This study
pGEX-*cdgC*	Plasmid derived from pGEX-4T-1 containing the ORF of *cdgC* gene, Ap^R^	This study
pCDR Δ*cdgC*	Plasmid derived from pCDR containing the 1,699 bp fragment (AB) in order to allow *cdgC* deletion by homologous recombination, Tc^R^	This study
pJB-*cdgC*	Plasmid derived from pJB3Tc20-Km^R^ containing the 1,089 bp fragment corresponding to *cdgC* gene and its native promoter	This study
pAZBR-*cdgC*	Derivative vector of pAZBR-T7-Gm carrying the 1089 bp fragment corresponding to *cdgC* gene and its native promoter among the chromosomal TWA69690.1 and TWA69689.1 putative proteins from *A. baldaniorum* Sp245 strain, Tc^R^, Gm^R^	This study

*Ap, Ampicillin; Km, Kanamycin; Tc, Tetracycline; Gm, Gentamycin; Cm, Chloramphenicol.*

To generate a *trans* genetic complementation construct, the ORF of the c*dgC* gene and its upstream intergenic region were amplified with the oligonucleotide pair Pr100206F/Pr100206R and cloned into the low-copy-number vector pJB3Tc20 (Blatny et al., 1997). Both the amplicon and the destination plasmid were digested with the REs *Eco*RI and *Kpn*I and ensembled using a T4 DNA ligase (Promega). The vector pAZBR-*cdgC* was generated to allow for *cis* genetic complementation of the Δ*cdgC* mutant strain by inserting *cdgC* and its promoter region in a neutral locus in the chromosome (Cruz-Pérez et al., 2021) of *A. baldaniorum* 59C. First, we constructed the pGEM-Gm^R^ plasmid by cloning into the pGEM-T Easy vector, the gentamycin resistance gene from the pBBR1MCS-5 vector (Kovach et al., 1994) which was amplified using the GmF and GmR primers. Next, we generated the suicide plasmid pAZBR-T7-Gm by subcloning the gentamycin resistance gene from pGEM-Gm^R^ into pAZBR-T7mCh (Cruz-Pérez et al., 2021) by digesting with the REs *Xho*I and *Spe*I. This plasmid enables the insertion of exogenous sequences through homologous recombination. The *cdgC* gene, and its native promoter were extracted from the plasmid pJB-*cdgC* using the REs *Sna*BI and *Eco*RI, this fragment was next cloned into pAZBR-T7-Gm. The pAZBR-*cdgC* construct was mobilized into *A. baldaniorum* 59C by biparental mating using *E. coli* S17.1 as a donor strain.

The pGEX-*cdgC* construct was generated to analyze the effect of the expression of *cdgC* on the c-di-GMP levels of the heterologous host *E. coli* S17.1. The *cdgC* gene was amplified with the *cdgC*-F and *cdgC*-R primers and the product was cloned into vector pGEM-T Easy generating pGEM-*cdgC*. The latter was digested with the REs *Eco*RI and *Sal*I and cloned into the expression plasmid pGEX-4T-1 generating pGEX-*cdgC*. The pGEX-*cdgC* plasmid was then used to transform *E. coli* S17.1 competent cells harboring the c-di-GMP biosensor pDZ-119 (Martínez-Méndez et al., 2021).

The pMP2450 vector containing the gene encoding the enhanced green fluorescent protein (eGFP) was constructed for competence and wheat root colonization assays. This plasmid was generated by cloning the kanamycin resistance cassette from the pJMS-Km (Ramírez-Mata et al., 2016) vector into the vector pMP2444 (Clontech). The kanamycin resistance cassette was extracted using the RE *Xba*I and cloned into a unique *Xba*I site in pMP2444 to generate pMP2450. All amplicons obtained for cloning were sequenced at the Sequencing Unit at the Universidad Nacional Autonoma de Mexico (UNAM). All strains generated in this study are listed in Table 1.

### Biofilm Formation Assays and Confocal Laser Scanning Microscopy

Biofilm formation was performed employing NFB^∗^ medium (C/N = 2) under static conditions, as previously described (Arruebarrena Di Palma et al., 2013), or under nitrogen fixation conditions using NFB medium. Briefly, a 3-day-old colony of *Azospirillum* strains (*A. baldaniorum*, *A. baldaniorum* 59C, *A. baldaniorum* C21, *A. baldaniorum* C87, *A. baldaniorum*-GFP, *A. baldaniorum* 59C-GFP, *A. baldaniorum* 2A65, and *A. baldaniorum* 2D8) grown in CR medium were inoculated into LB^∗^ medium and incubated at 30°C with shaking at 150 rpm until the culture reached an optical density at 600 nm (OD600) of 1.1–1.4. Cells were harvested by centrifugation (5,000 rpm), washed with phosphate buffer (66 mM) pH 7.0, and concentrated to an OD600 of 2. Biofilm formation was determined by the crystal violet assay as previously reported (Arruebarrena Di Palma et al., 2013). Alternatively, for CLSM, the cells were diluted at 1:100 in NFB^∗^ medium, and 3.9 mL of this suspension was transferred to a glass-bottom FluoroDish device (Thermo Fisher Scientific). Calcofluor-White colorant (CWC) (Sigma-Aldrich, United States) was added to a final concentration of 85 μM. Cultures were incubated under static conditions at 30°C for 5 days. Then, biofilm formation and exopolysaccharide production were evaluated with an inverted CLSM Nikon Eclipse Ti-E C2+ (Nikon Instruments, Tokyo, Japan) using a 60x Plan lambda objective. eGFP fluorescence excitation and emission were set at wavelengths of 488 and 509 nm, respectively. CWC was excited at 380 nm and its emission was acquired at a wavelength of 475 nm.

### Calcofluor-White Binding Assay

The binding of exopolysaccharides to calcofluor-white colorant (CWC) was performed as described previously (Spiers et al., 2003). Briefly, 3-day-grown colonies of *Azospirillum* strains were inoculated into LB^∗^ medium and incubated at 30°C with agitation at 150 rpm until the cultures reached an OD600 of 1.1–1.4. Cells were harvested by centrifugation (5,000 rpm), washed with phosphate buffer (66 mM) pH 7.0 and resuspended to a 2 OD600 biomass. Cells were diluted 1:100 in NFB^∗^ or NFB media with an initial OD600 of 0.1 (∼ 2 × 10^7^ CFU/mL), and 2 mL of this suspension were transferred to a flat bottom polystyrene well (Corning Incorporated) and incubated under static conditions at 30°C for 5 days. The supernatant of each well was removed and 1 mL NFB^∗^ or NFB supplemented with CWC at a concentration of 50 μg/mL was added. Cells were incubated at 30°C for 2 h under static conditions. Afterward, the media with CWC was replaced by 1 mL of 96% ethanol. Next, the content of each well was recovered and harvested by centrifugation at 13,000 rpm for 5 min. Then, μg/mL of CWC binding colorant to cells was determined in a standard curve and subsequently normalized with OD600 biomass (Spiers et al., 2003; O’Toole, 2010).

### Motility Assays

Motility assays were performed as previously described (Alexandre et al., 2000; Cruz-Pérez et al., 2021) with some modifications. Briefly, strains were grown in LB^∗^) and at 30°C and 150 rpm for 18 h. Next, a 5 μL drop containing approximately 5 × 10^6^ CFU/mL of each evaluated strain was spotted onto soft agar plates with K-minimal medium, agar 0.25% (w/v), and succinate, malate, pyruvate, proline, and fructose as the carbon source to be tested as an attractant to a final concentration of 10 mM. Plates were incubated under static conditions at 30°C for 24 and 48 h. Swimming rings were measured and statistically analyzed.

### Estimation of Cellular Levels of Cyclic-di-GMP

The analysis of c-di-GMP levels was done in *E. coli* S17.1 strains carrying the pGEX-*cdgC*/pDZ-119, pGEX-4T-1/pDZ-119, or pDZ-119 vectors, that were grown in LB medium supplemented with ampicillin at 100 μg/mL and/or chloramphenicol at 10 μg/mL. CdgA, a functional and characterized DGC from *A. brasilense* Sp7 (Ramírez-Mata et al., 2016), expressed from the pLIL-2 vector was used as a positive control to validate the assay. c-di-GMP accumulation was evaluated as reported by Zhou et al. (2016). Briefly, *E. coli* cultures were grown to an OD600 of 0.6, afterward IPTG was added to a final concentration of 0.01 or 0.1 mM to induce the expression of the DGCs. Induction was carried out at 30°C for 24 h with shaking at 150 rpm. Cultures were concentrated 10-fold and resuspended in water. c-di-GMP production was analyzed macroscopically relating color intensity with the production of the second messenger. Microscopic assessment of c-di-GMP was performed using a Nikon Eclipse TE2000U fluorescence microscope. A drop of the induced culture was deposited on a coverslip and covered with a 1% agarose plug. The excitation and emission of the calibrator AmCyan fluorophore were recorded at 457 and 520 nm, respectively. The reporter TurboRFP fluorophore was excited at 553 nm, while its emission was measured at 574 nm. Images obtained were edited with the Nikon NIS Elements software.

### Competition and Wheat Root Colonization Assays

The competition assay and root colonization experiments were performed as described previously (Ramirez-Mata et al., 2018). Briefly, wheat seeds (*Triticum aestivum*) of the “Nana” variety were surface sterilized with 70% ethanol for 1 min, 1% sodium hypochlorite for 30 min and a mix of cycloheximide, 150 μg/mL; streptomycin, 250 μg/mL; tetracycline, 20 μg/mL, and fluconazole, 150 μg/mL. Subsequently, the seeds were washed 4 times with sterile deionized water for 1 min, placed on Petri plates with agar 0.6% (w/v), and germinated for 2 days at 30°C. Seedlings were transferred into sterile glass tubes containing 15 mL of Hoagland hydroponic solution supplemented with 25 mM KNO_3_, maintained for 7 days, and placed into an environmental chamber at 80% relative humidity with a photoperiod of 14 h light at 25°C and 10 h dark at 16°C. Six plants for each strain were inoculated by dip 10^7^ CFU/mL *A. baldaniorum* 2449 or *A. baldaniorum* 2450; for the competition assay *Azospirillum* cells were inoculated to a 1:1 (5 × 10^6^ CFU/mL of each strain) (Ramírez-Mata et al., 2018). All inoculated plantlets were maintained under the above conditions for 7 days. Seven days postinoculation (dpi), the plant roots were macerated with PBS buffer (pH 6.8), and bacterial colonization was determined by colony counting using CR medium supplemented with Gm for 2449 strain or Km for 2450 strain. For competition assay bacteria were plated in RC medium without antibiotic, and were further growing in CR media supplemented with Km or Gm. Additionally, seedling inoculated root sections were examined using an inverted CLSM Nikon Eclipse Ti-E C2+ (Nikon Instruments, Tokyo, Japan) to evaluate the colonization patterns of these strains. Images obtained were edited employing NIS Elements software.

### Bacterial Endophytic Quantitation

To quantify bacterial internalization to wheat roots, an external disinfection protocol was implemented, as described above. Seedlings were transferred into sterile glass tubes containing 15 mL of Hoagland hydroponic solution supplemented with 25 mM KNO_3_ and without antibiotics. Seven-day-old plants were inoculated by dip with 10^7^ CFU/mL of *A. baldaniorum*-GFP, *A. baldaniorum* 59C-GFP, or *A. baldaniorum* 2A65 (Ramírez-Mata et al., 2018) and maintained as above for 7 days. Subsequently the post-inoculated roots were immersed in sodium hypochlorite (0.6%) for 15 s with subsequent washes using phosphate buffer pH 6.8. Roots were weighed out and macerated. Endophytic colonization of *A. baldaniorum*-GFP, *A. baldaniorum* 59C-GFP were quantified by CFU plating in CR plates supplemented with Gm and for *A. baldaniorum* 2A65 by CFU plating in CR plates supplemented with both Gm and Km.

### Statistical Analyses

Data obtained were analyzed by Student’s *t*-test to determine if a statistically significant difference existed between the examined strains. Differences were cataloged as statistically significant when *p*-values were < 0.05.

## Results

### Bioinformatic Analysis and Three-Dimensional Modeling

**FIGURE 1:**
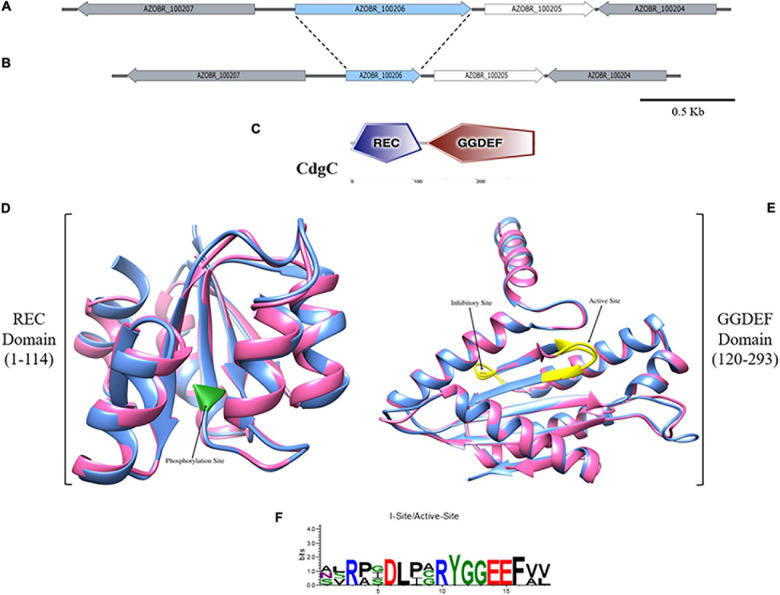
Domain-architecture and structure prediction of CdgC. **(A)** Genetic context of the AZOBR_100206 (marked in blue) gene which codifies for the CdgC protein. Downstream of it and separated by an intergenic region of 68 pb, AZOBR_100205 gene is found. **(B)** Scheme representation of the genetic context after the deletion of 509 pb belonging to the ORF of *cdgC* gene. This deletion leaded to the loss of the nucleotides that encode for the catalytic and inhibitory sites of the GGDEF, and the aspartate 51 located at the REC domain. Bar represents 500 pb. **(C)** Graphic representation of the domain architecture of CdgC obtained from the webserver SMART. **(D)** Superimposed ribbon diagrams from the REC domain of CdgC (pink) and the REC domain of WspR (blue) from *P. aeruginosa*. The residue involved in phosphorylation is marked in green. **(E)** Superimposed ribbon diagrams from the DGC domain of CdgC (pink) and the DGC domain of WspR (blue) from *P. aeruginosa*. Active and inhibitory sites are colored in yellow. Modeling of the REC and DGC domains of CdgC was carried out employing WspR as a model. REC domain (C-score: 1.38; TM-score: 0.91 ± 0.06; RMSD: 1.7 ± 1.4 Å), DGC domain (C-score: 1.71; TM-score: 0.95 ± 0.05; RMSD: 1.8 ± 1.5 Å). **(F)** Web Logo showing conserved inhibitory and active sites of CdgC compared to WspR.

*A. baldaniorum* contains 20 genes that encode putative proteins with homology to DGC proteins (Ramirez-Mata et al., 2018). In this work, we focus on a DGC encoded by the AZOBR_100206 gene, located on the bacterial chromosome (Wisniewski-Dyé et al., 2011). The AZOBR_100205 gene encoding a putative Nudix hydrolase, is located immediately downstream of AZOBR_100206 (Figures 1A,B). The analysis of the domain architecture of this protein of 294 amino acids in length revealed the presence of two domains: a REC or receptor domain located at the N-terminus of the protein and a GGDEF domain at the C-terminus (Figures 1C,D and Supplementary Figure 1A). We named this DGC CdgC (WP_014240083.1). Our analysis revealed that CdgC has a domain architecture that resembles that of WspR, a key regulator of exopolysaccharide production in *P*. *aeruginosa* (Güvener and Harwood, 2007; Ganesh and Rai, 2018). We speculated that CdgC could play similar roles in processes associated with surface attachment and biofilm formation in *A. baldaniorum*. We further analyzed the conservation pattern of amino acid motifs important for domain function, using WspR and PleD as reference sequences (Güvener and Harwood, 2007; Malone et al., 2007). The REC domain of CdgC spans amino acids from position 1 to 114, with the putative phosphorylation site being an aspartate residue at position 51 (Supplementary Figures 1A,B). This aspartate residue aligns with aspartate residues 70 and 52 from WspR and PleD, respectively, which have been proposed as the phosphorylation targets in these response regulators (Güvener and Harwood, 2007; Wassmann et al., 2007; Figure 1D). The GGDEF domain spans amino acid residues from position 120 to 293. The GGEEF amino acid residues involved in catalysis, were located in positions 210–214, while and inhibitory site (I-site) that matches the consensus RXXD was found in positions 201–204 (Chan et al., 2004; Vorobiev et al., 2012; Figures 1E,F). The catalytic site of CdgC (Gly^210^, Gly^211^, Glu^212^, Glu^213^, Phe^214^), is located between the β_2_ and β_3_ strands. The I-site (Arg^201^, Pro^202^, Gly^203^, Asp^204^) is located five amino acids upstream of the GGEEF motif (Supplementary Figure 1B). It is noteworthy that both WspR and PleD have a GGEEF motif and an I-site. The GTP-interacting and additional catalytic residues observed in previous DGC domain structures (Malone et al., 2007; Römling et al., 2013, 2017) are conserved in CdgC, strongly supporting that this DGC might be active.

CdgC showed 33.33, 25, and 25.56% identity with the first REC and second REC domains of PleD and the REC domain of WspR, respectively (Supplementary Figure 1A). On the other hand, the DGC domain of CdgC showed 41 and 39.66% identity with the DGC domains of the PleD and WspR proteins, respectively (Supplementary Figure 1B). The secondary structure of the C-terminal domain is made from five α-helices and seven short β-strands with a sequence α1–α2–β1–α3–β2–β3–α4–β4–β5–β6–α5–β7 with the GGDEF motif located within a β-hairpin between β2 and β3, these features are conserved in the response regulators WspR and PleD (Figure 1E and Supplementary Figure 1B).

Three-dimensional structural analysis of the CdgC protein was performed by comparison with the crystal structure of WspR (PDB number access 3BRE). The superposition of the REC (C-score 1.38) domain of CdgC with the REC domain of WspR (Figure 1D) revealed a similar spatial distribution. Whilst the modeling of the DGC domain of CdgC (C-score 1.71) proved to be constituted by five α helices and seven β-strands (Figure 1E), it is worth mentioning that a C-score close to 2 gives the model greater reliability. In addition, the active site showed the presence of glycines, which occur in the corresponding position for the substrate GTP in the predicted structure of the GGEEF domain protein that is conserved and provides space for ribosyl sugars and phosphates, thus explaining the conservation of these residues, as shown in the Web-logo model (Figure 1F). Comparison with previously solved DGC structures shows a similarity of the active and inhibitory sites; the latter is characterized by an RXXD sequence, and both are conserved, similar to WspR and PleD, the most well-investigated diguanylate cyclases (Chan et al., 2004; Römling et al., 2017).

### Biofilm Formation and Exopolysaccharide Production Are Subject of Regulation by CdgC

To evaluate if CdgC, like WspR and PleD, participates in physiological processes involved in surface attachment and biofilm formation we first generated a variant strain of *A. baldaniorum* that is isogenic except for the deletion of *cdgC* (*A. baldaniorum* 59C). Additionally, two complemented variants were generated, one where *cdgC* was inserted into the chromosome of *A. baldaniorum* 59C (*A. baldanio*rum C21) and another one where *cdgC* was expressed exogenously from a plasmid (*A. baldaniorum* 3A13).

We first analyzed the growth rate in liquid media of the 59C mutant strain and the complemented variants (Supplementary Figure 2). Our results showed that the strains exhibited similar growth characteristics. Next, to evaluate biofilm architecture, we mobilized into the parental strain, the mutant strain *A. baldaniorum* 59C, and the complemented strain *A. baldaniorum* 3A13, a plasmid expressing the fluorescent protein eGFP (*A. baldaniorum*-GFP, *A. baldaniorum* 59C-GFP, and *A. baldaniorum* 2A65 strains, respectively).

**FIGURE 2:**
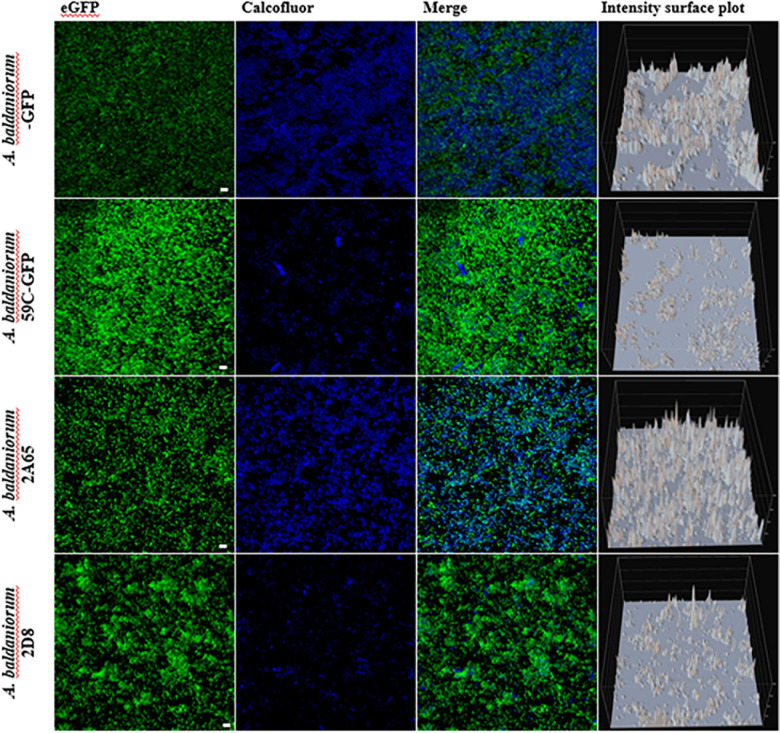
Biofilm formation analysis with confocal microscopy. Maximum projection confocal laser scanning microscopy images of 5-day old static biofilms grown in NFB media and tagged with an eGFP fluorescent protein (shown in green) and dyed with calcofluor CWC (shown in blue). The *A. baldaniorum* WT, *A. baldaniorum* 59C mutant, *A. baldaniorum* C21 mutant complemented with *cdgC* gene, *A. baldaniorum* C87 mutant without *cdgC* gene strains. Scale bar 10 μm. Intensity surface plot corresponding to the emitted fluorescence by CWC is shown in the last panels. The Nikon Eclipse Ti-E C2+ (CLSM) was used with a 60x Plan lambda objective. These are representative images from at least three independent experiments.

Biofilms were grown under denitrification or nitrogen fixation (NFB) conditions for 5 days, stained with CWC, and visualized employing an inverted CLSM. The CWC fluorophore binds to compounds with β-1,3 and β-1,4 bonds and it has been used to detect exopolysaccharides (Del Gallo et al., 1989). Data obtained from confocal microscopy showed no difference between wild-type, mutant, and complemented strains when strains were grown under denitrification conditions (minimal medium plus KNO_3_). The three-dimensional architecture of the biofilms (Z-projection of x-y stacks with 20.1 μm, optical sections) showed similar thicknesses (Supplementary Figures 3A–C). In contrast, in cells growing under nitrogen-fixing conditions (NFB) a considerable alteration in exopolysaccharides stained with CWC was observed in the mutant strain, showing a decrease in the production of a calcofluor-binding polysaccharide compared to the wild-type strain, and this deficiency was complemented by exogenously expressing *cdgC* (Figure 2). Furthermore, we observed severe defects in biofilm structure in the Δ*cdgC* mutant strain compared to the wild-type strain (Figure 2). These observations suggest that CdgC is important for exopolysaccharides production in biofilms and that biofilms grown under nitrogen-fixing conditions are more sensitive to the absence of CdgC.

**FIGURE 3:**
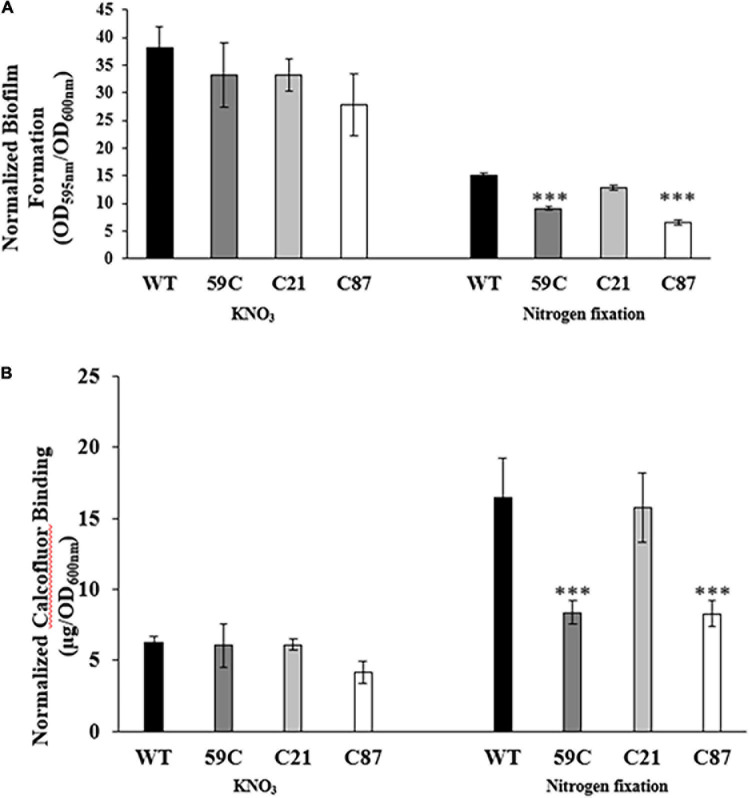
Biofilm formation and exopolysaccharide production assays. **(A)** Bar plots representing the mean and standard deviation of biofilm quantification assays of *Azospirillum* strains (*A. baldaniorum* WT, *A. baldaniorum* 59C mutant, *A. baldaniorum* C21 mutant complemented with *cdgC* gene, *A. baldaniorum* C87 mutant without *cdgC* gene) grown in NFB* media supplemented with KNO_3_ or NFB media. **(B)** Bar plots representing the mean and standard deviation of calcofluor-binding assays from same above *Azospirillum* strains grown in NFB* media supplemented with KNO_3_ or NFB media. The data shown were obtained from three independent experiments. The means of the wild-type strain and the mutant strain were compared using a Student’s *t*-test. Asterisks indicate a *P-*value = 0.00042 in **(A)** and *P*-value = 0.00868 in **(B)**.

To quantify biofilm formation, we used the crystal violet staining method. Our results revealed that biofilm formation is more proficient under denitrification growth conditions compared to nitrogen-fixing conditions (Figure 3A). Similar to our observations regarding biofilm structure, we did not observe differences in biofilm formation under denitrification conditions in any of the analyzed strains. In contrast, we did observe a reduction in biofilm formation in the mutant strain *A. baldaniorum* 59C and *A. baldaniorum* C87 (control mutant strain), which was complemented by a single copy of *cdgC* inserted into the chromosome (*A. balda*niorum C21) (Figure 3A).

Together, these results strongly suggest that CdgC plays an important role in biofilm formation especially under conditions where the nitrogen source is obtained through nitrogen fixation. The defects on biofilm formation observed in the mutant strain *A. baldaniorum* 59C, are likely linked to an impairment in exopolysaccharides production.

### CdgC Is Not a Regulator of Motility

Motility and biofilm formation are typically inversely regulated by the second messenger c-di-GMP (Ryjenkov et al., 2006; Ramírez-Mata et al., 2016; Little et al., 2019). Hence, we analyzed if the absence of *cdgC* affects the motility of *A. baldaniorum* in soft agar plates. Our results did not reveal differences in motility for any of the strains analyzed. We speculate that this could be due to compensatory effects of several other genes encoding DGCs found in the genome of *A. baldaniorum* (Supplementary Figure 4).

### CdgC Promotes Cyclic-di-GMP Accumulation in an Heterologous Host

**FIGURE 4:**
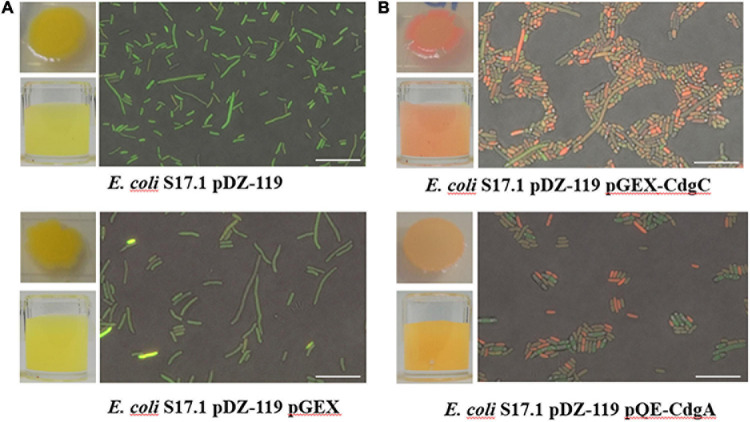
c-di-GMP accumulation assay using a specific biosensor. Representative images of visible fluorescence of concentrated cultures and microscopic fluorescence detection in individual cells in **(A)** control strains *E. coli* S17.1 pDZ-119 and *E. coli* S17.1 pDZ-119/pGEX; **(B)** strains overproducing CdgC or CdgA. The microscopic assessment of c-di-GMP levels was performed using a Nikon Eclipse TE2000-U fluorescence microscope. The excitation and emission of the AmCyan fluorophore were recorded at 489 and 519 nm, respectively. The TurboRFP fluorophore was excited at 553 nm, and emission was measured at 574 nm. Images obtained were edited with Nikon NIS Elements software. Bar 10 μm. The data presented are representative of three independent experiments.

As mentioned above, CdgC has all the structural and sequence elements of active diguanylate cyclases (Figure 1). To demonstrate that CdgC can promote c-di-GMP accumulation we coexpressed it with a c-di-GMP biosensor in the heterologous host *E. coli* S17.1. The usefulness of this c-di-GMP biosensor to measure relative abundance of c-di-GMP has been previously reported (Zamorano-Sánchez et al., 2019; Wu et al., 2020; Martínez-Méndez et al., 2021). This biosensor produces two fluorescent proteins, AmCyan and TurboRFP, both proteins are under the control of a constitutive promoter. However, the production of TurboRFP is regulated by two c-di-GMP riboswitches located in tandem upstream of the *turborfp* gene. These riboswitches prevent production of TurboRFP when the levels of c-di-GMP are low (Zamorano-Sánchez et al., 2019; Wu et al., 2020; Martínez-Méndez et al., 2021). Under the conditions tested, most of the *E. coli* S17.1 pDZ-119 cells expressing the c-di-GMP biosensor showed green fluorescence, indicating that mostly the AmCyan fluorophore is being produced and that c-di-GMP levels are very low in this strain (Figure 4A). The additional presence of the empty expression plasmid pGEX did not affect c-di-GMP levels (Figure 4A). In contrast, expression of either *cdgC* or *cdgA* from an IPTG inducible promoter in pGEX-CdgC and pQE-CdgA, respectively, resulted in an increase in red fluorescence likely due to elevated production of TurboRFP and c-di-GMP compared to the control strains (Figure 4B). The CdgA protein was used as a positive control for the production of c-di-GMP since its function as a DGC was previously characterized (Ramírez-Mata et al., 2016). These data strongly suggest that CdgC is a functional DGC capable of producing c-di-GMP.

### CdgC Is Required for Optimal Wheat Root Internalization

**FIGURE 5:**
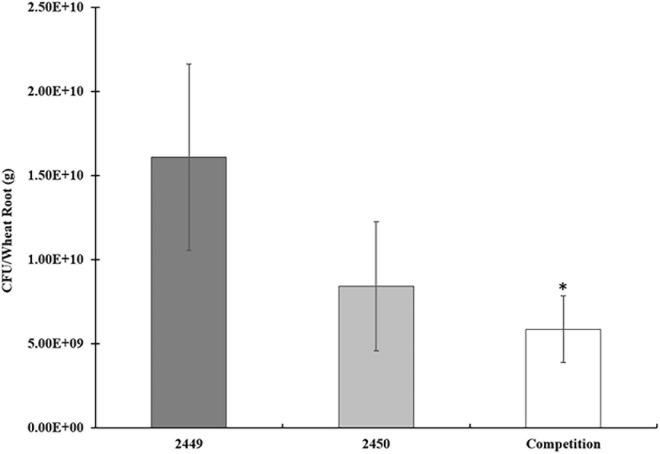
Competition assays in wheat roots: *A. baldaniorum* 2449 WT strain, *A. baldaniorum* 2450 mutant strain, and competition assay seedling inoculated with 1.1 with both strains, as described in “Materials and Methods” section. Bar plots representing the mean and standard error of colony-forming units recovered per gram of wheat root from colonization assays. The data was obtained from three independent experiments. The mean of WT and the competition assay was compared with a Student’s *t*-test and was significantly different with a *P-*value = 0.0142 indicated with one asterisk.

Since we showed that CdgC is required for exopolysaccharides production we next asked if its absence affects bacterial colonization of wheat roots. We speculated that alterations in colonization patterns might be observed in single-strain infections or competition assays. To test these possibilities, we tagged the wild-type strain with the mCherry protein (*A. baldaniorum* 2449) and the Δ*cdgC* mutant strain with the eGFP protein (*A. baldaniorum* 2450). The number of bacteria colonized (CFU/mL) to wheat roots was determined 7 days post-inoculation for the individual strains and the competition assay. The number of cells colonizing the roots was not statistically different between strains 2449 and 2450, however, we observed reduced root-colonization of cells in the competition assay (Figure 5). The data indicated that the WT-2449 and (59C-2450)-tagged strains were able to grow as individual infections at the same level (1.6 × 10^10^ /g wet root and 8.4× 10^9^ /g root, respectively) colonizing the wheat plant roots well. However, when a competition assay was performed inoculating both strains with the same proportion of CFU/mL (1:1), a slight decrease in mutant 59C-2450 and WT-2449 bacteria colonizing the wheat roots was observed, with a statistically significant difference when two strains occur in a mixed infection (5.8 × 10^9^/g wet root). These data would reflect that the mutant strain is able to grow as efficiently as the wild type (Figure 5) and that the presence of the mutant strain upsets the growth of the wild-type strain, which might suggest an effect of the mutant (59C-2450) on the growth of WT-2449 into wheat roots.

**FIGURE 6:**
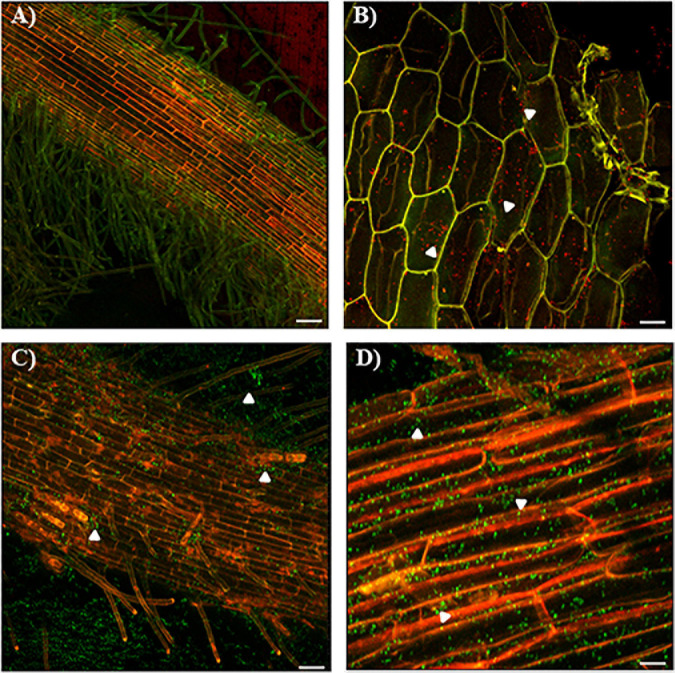
Confocal images of colonization of wheat roots by CLSM. **(A)** Colonization pattern of the mCherry-tagged *A. baldaniorum* 2449 in wheat roots after 7 days of inoculation. Typical endophytic behavior of the wild-type strain. **(B)** Wild-type strain (red fluorescence) location was corroborated through a longitudinal cut of wheat root. White arrowheads indicate mCherry-expressing *A. baldaniorum* 2449 strain. The images **(A,B)** represent the merge of two micrographs of mCherry detection (shown in red) and plant autofluorescence (shown in green). **(C)** Colonization pattern of the eGFP-tagged *A. baldaniorum* 2450 in wheat roots after 7 days of inoculation, showing an alteration in its ability to internalize, locating outside of wheat root. **(D)** Surface colonization pattern of *A. baldaniorum* 2450 expressing eGFP in wheat roots after 7 days of inoculation, indicated with arrowheads. Magnification 10X. The images **(C,D)** represent the merge of two micrographs of eGFP detection (shown in green) and plant autofluorescence (shown in red). Bars represents 50 μm. Representative images were acquired by examination of several images with a 20X objective, and 1.8 amplification zoom.

To further evaluate the process of infection of the wild-type strain and the Δ*cdgC* mutant strain we analyzed the bacterial-plant association using confocal microscopy (Figure 6). The wild-type strain *A. baldaniorum* 2449 and the mutant strain *A. baldaniorum* 2450 showed distinct colonization patterns (Figure 6). *A. baldaniorum* 2449 showed the typical endophyte colonization pattern showing bacteria (in red fluorescence) internalized in plant (Figures 6A,B) cells. In contrast, the *A. baldaniorum* 2450 strain remained on the root surface, forming dispersed bacterial aggregates (green fluorescence) (Figures 6C,D). These results suggest that the absence of CdgC renders the cells unable to internalize efficiently into wheat roots.

**FIGURE 7:**
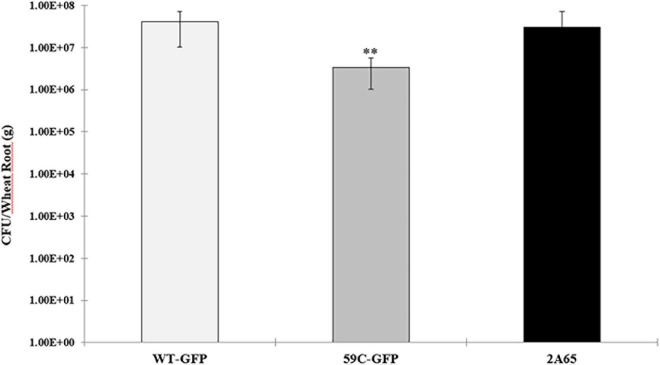
Endophytic colonization of *A. baldaniorum* and derivative strains: *A. baldaniorum* –GFP-Gm^R^, WT strain, *A. baldaniorum* 59C-GFP-Gm^R^, mutant strain, and *A. baldaniorum* 2A65-GFP-Gm^R^, Km^R^, complemented strain. Bar plots representing the mean and standard error of colony-forming units recovered per gram of wheat root after surface-sterilization (endophytic bacteria). The data was obtained from three independent experiments. The mean of WT and the 59C mutant strain assay was compared with a Student’s *t*-test and was significantly different with a *P*-value = 0.004011 and indicated with asterisks.

Finally, to corroborate the data obtained by confocal microscopy, we quantified the endophytic bacteria of colonized wheat roots. As expected, the mutant strain showed an alteration in its capability to internalize to wheat roots compared to the wild-type and complemented strains (Figure 7).

## Discussion

The formation of biofilms enables bacteria to survive and propagate despite the presence of toxic compounds or other external stresses, and *A. baldaniorum* is no exception. This bacterium adopts two different lifestyles—the motile form and the sessile form (Lerner et al., 2009; Shelud’ko et al., 2010; Wang et al., 2017; Ramirez-Mata et al., 2018). Bacterial colonization of the host surfaces is a prerequisite for tissue invasion. To promote a long and successful interaction, bacteria tend to form biofilms. Biofilms allow bacteria to survive nutrient limitations, fluctuations in oxygen levels, and massive changes in osmolarity (Bible et al., 2015; Krol et al., 2020). Moreover, high levels of c-di-GMP promote biofilm formation, while low intracellular levels induce a planktonic lifestyle (Wang et al., 2019). In this work, we explored the role of the *cdgC* gene from *A. baldaniorum* in the formation of biofilms and colonization of seedling wheat roots.

The computational prediction of the structure of the c-di-GMP metabolizing enzyme CdgC revealed that it is composed of a receptor (REC) domain located at the N-terminus and a diguanylate cyclase (DGC) domain at the C-terminus (Figure 1). REC domains are typically phosphorylated, in the case of DGCs such as PleD and WspR this leads to a conformational change that causes the activation of the GGDEF domain (Paul et al., 2004; De et al., 2008). We analyzed the genomic context of AZOBR_100206 encompassing 14 Kbs upstream and downstream of the structural gene, we did not found genes encoding for histidine kinases that could be the potential cognate of CdgC. The genome of the *A. baldaniorun* (formerly named *A. brasilense* strain Sp245) has 166 putative histidine kinases in the genome (Borland et al., 2015), many of which are considered orphan due to the absence of a response regulator in their vicinity. It is possible that an orphan histidine kinase regulates the phosphorylation state of CdgC, this potential regulatory pair and the relevance of phosphorylation for the activity of CdgC will be important aspects to analyze in depth in future work. The REC of CdgC is composed of 114 amino acids that acquire the characteristic (β/α)_5_-fold of these domains, while its active site consisting of three aspartates, a lysine, and a threonine, is fully conserved. In the case of the REC domain of CdgC and according to the canonical protein PleD, aspartates D_13_ and D_14_ may be involved in magnesium binding, and aspartate D_5__7_ may be considered the site of phosphorylation. Finally, threonine T_8__7_, phenylalanine F_10__6_, and lysine K_10__9_ could be implicated in structural changes once the domain is phosphorylated (Lee et al., 2001; Chan et al., 2004; Figures 1C,D and Supplementary Figure 1A). The diguanylate cyclase domain is constituted of 173 amino acids. CdgC shares the characteristic (α/β)_5_ core of diguanylate cyclase enzymes (Zähringer et al., 2013; Deepthi et al., 2014). According to the secondary and tridimensional model (Figure 1E), the CdgC model showed a similar spatial distribution regarding both proteins previously mentioned, which suggests the putative function of CdgC.

Next, to corroborate the predicted role of the *cdgC* gene by computational study, in line with this hypothesis, we analyzed its contribution to the *Azospirillum*-wheat root interaction, which starts with motility toward plant roots by the polar flagellum after primary and weak attachment toward roots mediated by bacterial surface proteins, capsular polysaccharides, and polar flagella (Egorenkova et al., 2001; Fukami et al., 2018; Viruega-Góngora et al., 2020). The second attachment, which is tight and irreversible, is ruled mainly by capsular polysaccharides, bacterial surface proteins, and exopolysaccharides that favor cell-to-cell adhesion (Rodríguez-Navarro et al., 2007; Wheatley and Poole, 2018). However, in this study, no significant changes were observed in motility, as visualized by swimming soft agar plates in minimal medium supplemented with some chemoattractants (Supplementary Figure 4), possibly because of functional redundancy in that the function of other genes encoding DGC sibling proteins might compensate for the diminished cellular level of c-di-GMP. Indeed, three other genes encoding DGC (GenBank accession numbers: AWJ90840.1, AWJ88319.1, and AWJ91498.1) with identities of 40, 38, and 42%, respectively, to CdgC (Supplementary Figure 5), which showed similar characteristic structures as has been described to be present in the sequenced genome of *A. baldaniorum* (Ramirez-Mata et al., 2018) might be responsible for the first baseline level of c-di-GMP production, resulting in a temporal inhibition of flagellar torque generation (Russell et al., 2013; O’Neal et al., 2019), which in a long-term assay was not determined. It is possible that using a real-time analysis to test the behaviors of wild-type and mutant strains to link chemotaxis responses to distinct chemicals identified in root exudates of wheat, we could observe the chemotactic temporal motile response of mutant Δ*cdgC* cells, which were not tested in this study (O’Neal et al., 2020). In addition, as motility assays were determined using a minimal medium supplemented with NH_4_Cl as nitrogen source an alternate explanation may be that the pathway mediated by CdgC is not activated under conditions of nitrogen supply. Further studies to generate double or triple mutants in genes encoding DGCs showing similar structures, and growing under NFB conditions, will be required.

It is well established in different bacteria that increased c-di-GMP levels are associated with enhanced production of exopolysaccharides followed by the transition of bacteria from motile and planktonic to sessile lifestyles (Römling et al., 2013; Pérez-Mendoza and Sanjuán, 2016; Valentini and Filloux, 2016). In agreement, we found that exopolysaccharide production was decreased 2.1-fold in the 59C mutant compared with the wild type, and the production was re-established in the complemented C21 strain when cells were grown in NFB medium (Figure 3B). Accordingly, we observed that the *cdgC* gene is involved in the formation of biofilms and exopolysaccharide production under nitrogen fixation (Figures 2, 3B), as previously outlined in *A. brasilense* Sp7 and *Pseudomonas stutzeri* (Bible et al., 2015; Wang et al., 2017; Shang et al., 2021). The role of the second messenger c-di-GMP levels in cells grown under nitrogen-fixing conditions has been described in *A. brasilense*, where the PII protein, which is a global regulator of nitrogen fixation, additionally mediates the control of homeostasis at the cell level of c-di-GMP *in vivo*, modulating cell motility, aerotaxis, and adherence behaviors, which are regulated through binding with two enzymes involved in the biosynthesis of c-di-GMP, albeit the genetic and biochemical mechanisms involved in this regulation have not yet been described (Gerhardt et al., 2020). Biofilm formation in cells grown in NFB^∗^ medium supplemented with KNO_3_ as a nitrogen source (Figure 3A), was not affected by the absence of CdgC. This is likely due to the compensatory function of other genes encoding DGCs, or by a masking effect that results from the production of NO and its positive effect on biofilm formation as was previously reported (Arruebarrena Di Palma et al., 2013).

The exopolysaccharides present in *Azospirillum* have affinity for lectins present in plants, favoring the interaction between both organisms. This union triggers changes in bacterial metabolism, such as an increase in atmospheric nitrogen fixation, excretion of ammonium, production of indole-3-acetic acid, stress endurance, and biofilm formation (Egorenkova et al., 2001; Lerner et al., 2009; Alen’kina et al., 2014; Wang et al., 2017). Additionally, an alteration in capsule composition has been demonstrated to drive a decrease in adsorption of *A. brasilense* toward wheat roots (Skvortsov and Ignatov, 1998; Egorenkova et al., 2001). Similarly, c-di-GMP has been shown to regulate rhizosphere colonization in beneficial plant bacteria, such as *Pseudomonas putida*, *Pseudomonas fluorescens*, and species of the genus *Rhizobium* (Pérez-Mendoza et al., 2014). Our results revealed that CdgC is involved in the process of incorporation of bacteria into root colonization (Figure 5). In *P. fluorescens* SBW25, the individual deletion of three putative DGCs produced a marginal decrease in bacterial attachment toward the root surface (Lerner et al., 2009; Little et al., 2019). Competitive fitness for attachment to wheat roots of the mutant strain 59C to the wild-type strain was obtained. Under such conditions, the mutant strain was found to be slightly affected in the colonization of wheat roots, thus revealing that only cumulative disruption genes encoding DGCs must be achieved to significantly decrease the multiplication of the bacterium in plant (Figure 5).

The molecular mechanisms of plant recognition, attachment, penetration, and endophytic colonization of *A. brasilense* are poorly understood. The polar flagellum of *A. brasilense* plays an important role in adsorption to plant roots (Croes et al., 1993), and bacterial exopolysaccharides are usually responsible for attachment to solid surfaces and other bacterial cells, thus forming microscopic and macroscopic cell aggregates after the biofilm matrix is constructed (Michiels et al., 1990; Burdman et al., 2000; Compant et al., 2010; Balsanelli et al., 2014; Pérez-Mendoza and Sanjuán, 2016). Interestingly, our results showed that the DGC CdgC is required for optimal biofilm formation and exopolysaccharide production, its presence does not influence motility, but it did affect root internalization under the conditions tested. This could suggest that surface attachment and perhaps other processes involved in tissue invasion such as the production of cell wall-degrading enzymes might be regulated by c-di-GMP through the DGC CdgC in *A. baldaniorum.* Together, our results showed that CdgC is an important contributor to the development of the bacterial-plant interaction between *A. baldaniorum* and wheat. It remains to be shown how this DGC, and c-di-GMP in general, control the process of infection and endophytic colonization of roots in this important PGPR species.

## Conclusion

In summary, our data suggest that the *cdgC* gene participates, at least partially, in the production of certain exopolysaccharides with affinity for the calcofluor dye and that their effect depends on the nitrogen source and is more significant for nitrogen fixation than under nitrate assimilation conditions. Here, we provide evidence that *A. baldaniorum* 59C cells likely have changed their cellular surface and respond by modifying endophytic colonization. This result also indicated that the *A. baldaniorum* and *A. baldaniorum* 3A13 strains differ in competitiveness in their ability to infect events. In addition, further studies will be conducted to demonstrate how c-di-GMP levels are regulated so that several DGC enzymes could participate differentially during the stages of rhizosphere colonization. More experiments need to be developed to understand the complete landscape of molecular determinants involved in the signaling cascade that responds to the levels of c-di-GMP in *Azospirillum*.

## Data Availability Statement

The datasets presented in this study can be found in online repositories. The names of the repository/repositories and accession number(s) can be found in the article/Supplementary Material.

## Author Contributions

BB conceived the project. DS and AR-M designed experiments. DS, DZ, MX-V, AR-M, and VV carried out experiments. DS, MX-V, AR-M, and DZ analyzed the data and prepared the figures and tables. DS wrote the draft manuscript. BB, DZ, and AR-M revised the manuscript. All authors read and approved the submission for publication.

## Conflict of Interest

The authors declare that the research was conducted in the absence of any commercial or financial relationships that could be construed as a potential conflict of interest.

## Publisher’s Note

All claims expressed in this article are solely those of the authors and do not necessarily represent those of their affiliated organizations, or those of the publisher, the editors and the reviewers. Any product that may be evaluated in this article, or claim that may be made by its manufacturer, is not guaranteed or endorsed by the publisher.
