# Supplementary material for: CdgC, a Cyclic-di-GMP Diguanylate Cyclase of Azospirillum baldaniorum Is Involved in Internalization to Wheat Roots
Source: Front Plant Sci. 2021 Oct 20;12:748393. doi: 10.3389/fpls.2021.748393 (PMC8564387; doi:10.3389/fpls.2021.748393)
Supplement: Supplementary file 1 [file Data_Sheet_1.docx]

Supplementary Material

CdgC, a Cyclic-di-GMP Diguanylate Cyclase of *Azospirillum baldaniorum* is Involved in the Internalization to Wheat Roots

Daniel Sierra Cacho^1^, David S. Zamorano Sánchez^2^, Ma. Luisa Xiqui-Vázquez^1^, Victor Iván Viruega Góngora^1^, Alberto Ramírez-Mata^1^, and Beatriz E. Baca^1^*

^1^Centro de Investigaciones en Ciencias Microbiológicas, Benemérita Universidad Autónoma de Puebla, Ciudad Universitaria, Puebla, Mexico

^2^Programa de Biología de Sistemas y Biología Sintética, Centro de Ciencias Genómicas, Universidad Nacional Autónoma de México, Cuernavaca Morelos, Mexico.

Corresponding Author
*Beatriz Eugenia Baca beatriz.baca@correo.buap.mx; beatrizebaca@gmail.com

## Supplementary Figures


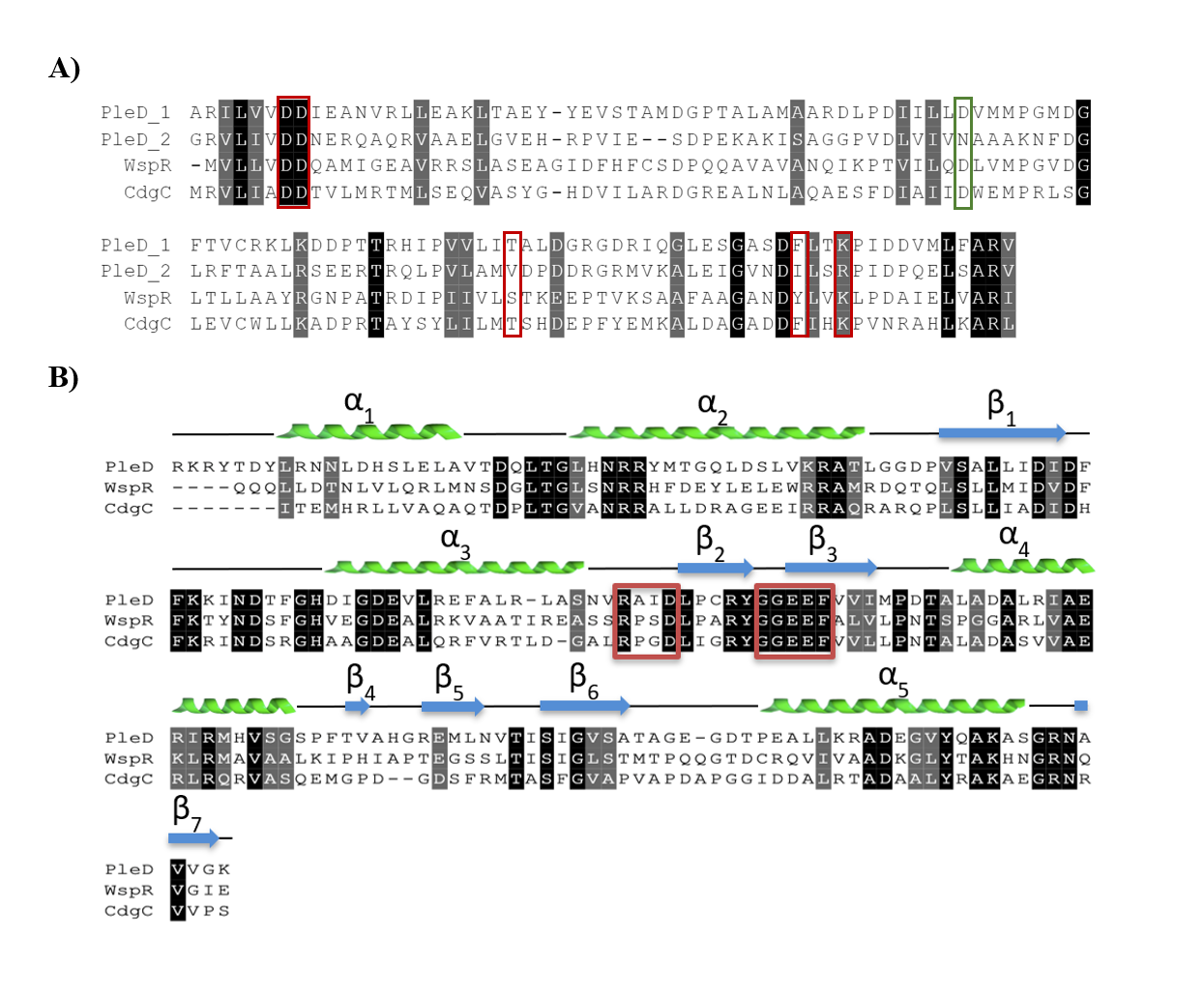


**Figure S1. Sequence alignment and comparison of REC and DGC domains of CdgC. A)** Sequence alignment of REC domain of CdgC. The aspartic acid (D_51_) receptor of phosphorylation is enclosed in a green box, whilst D_7_, D_8_, T_81_, F_100_, and K_103_ are encased in red boxes. **B)** Sequence alignment of DGC domains. The characteristic inhibitory (201-204) and active (210-214) sites are encased in red boxes. Active site of CdgC is fully conserved. Green helices represent α-helices; blue arrows represent β-strands. Inhibitory site (RxxD) and active site (GGEEF) are enclosed by red squares. Active motif is located between β_2_ and β_3_ strands. Primary sequence of CdgC was compared against the characterized DGCs PleD from *C. crescentus* and WspR from *P. aeruginosa*. Clustal Omega software was employed to perform the alignment. Identical or similar residues are showed in a black or gray background, respectively (4, 6-8, 9-10).

**
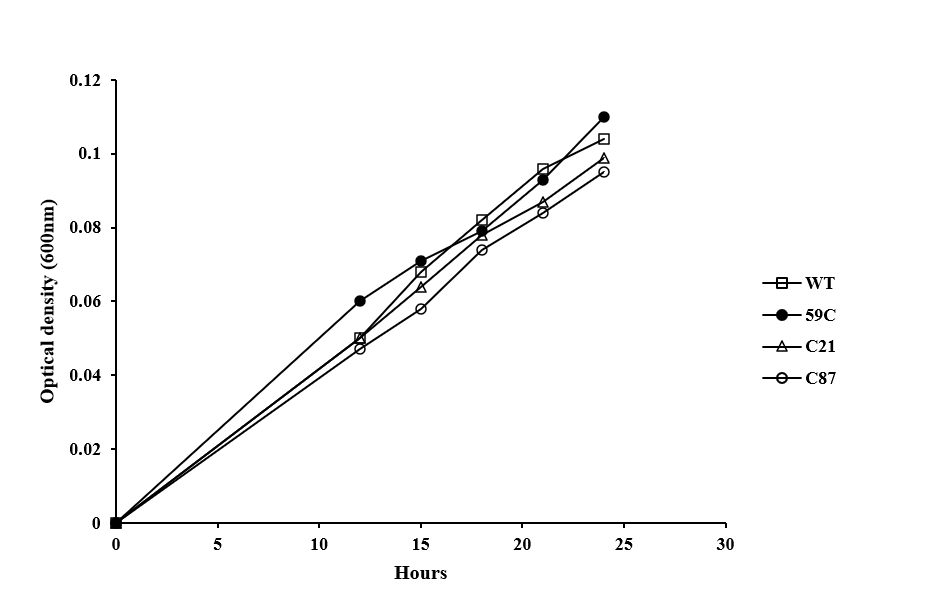
**

**Figure S2. Growth curve of *A. baldaniorum*, *A. baldaniorum* 59C, *A*. *baldaniorum* C21 and *A. baldaniorum* C87.** Growth curve of analyzed *Azospirillum* strains were done using NFB media (1). Growth was measured at OD_600nm_ every three hours until signs of flocculation were observed. *Azospirillum* strains (*A. baldaniorum*, WT; Δ*cdgC* mutant, 59C; complemented strain, C21; control strain with the empty vector, C87). Data showed is representative from three independent cultures of each strain.


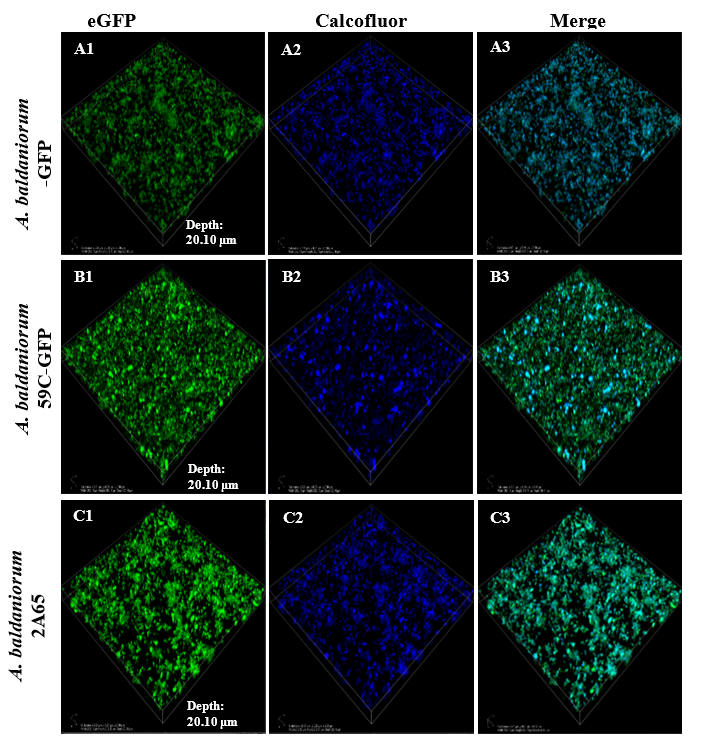


**Figure S3. Three-dimensional architecture of biofilm.** Biofilm formation assays were performed employing the eGFP-tagged strains (A1, B1, C1) and with the addition of calcofluor white colorant (A2, B2, C2) in order to exhibit the exopolysaccharide component of biofilm matrix. *Azospirillum* strains (*A. baldaniorum*-GFP, WT; *A. baldaniorum* 59C-GFP, mutant strain; *A. baldaniorum* 2A65, complemented with *cdgC* gene). Representative top views of 5 days biofilm grown in NFb media supplemented with KNO_3_.


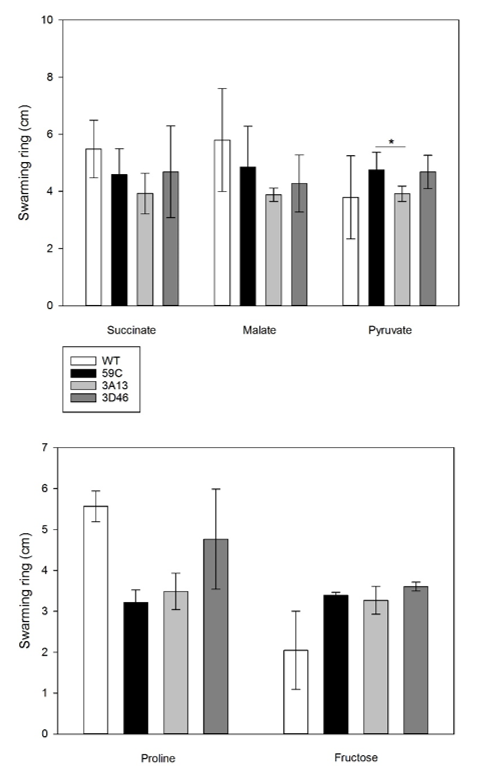


**Fig. S4. Motility of the *A. baldaniorum*, its Δ*cdgC* mutant, and complemented strain**. Assays were performed using soft agar [0.25%] and inoculating a 5 μl drop of a bacterial suspension containing 1x10^5^-5.5x10^6^ CFU. Swimming ring was measured 48 hours post inoculation (2,3). Standard deviations of replicates are represented by the error bars. Asterisk represents significant difference (*P* < 0.05). WT: *A. baldaniorum*; 59C: Δ*cdgC* mutant; 3A13: complemented strain; 3D46: control strain with the empty vector.

**
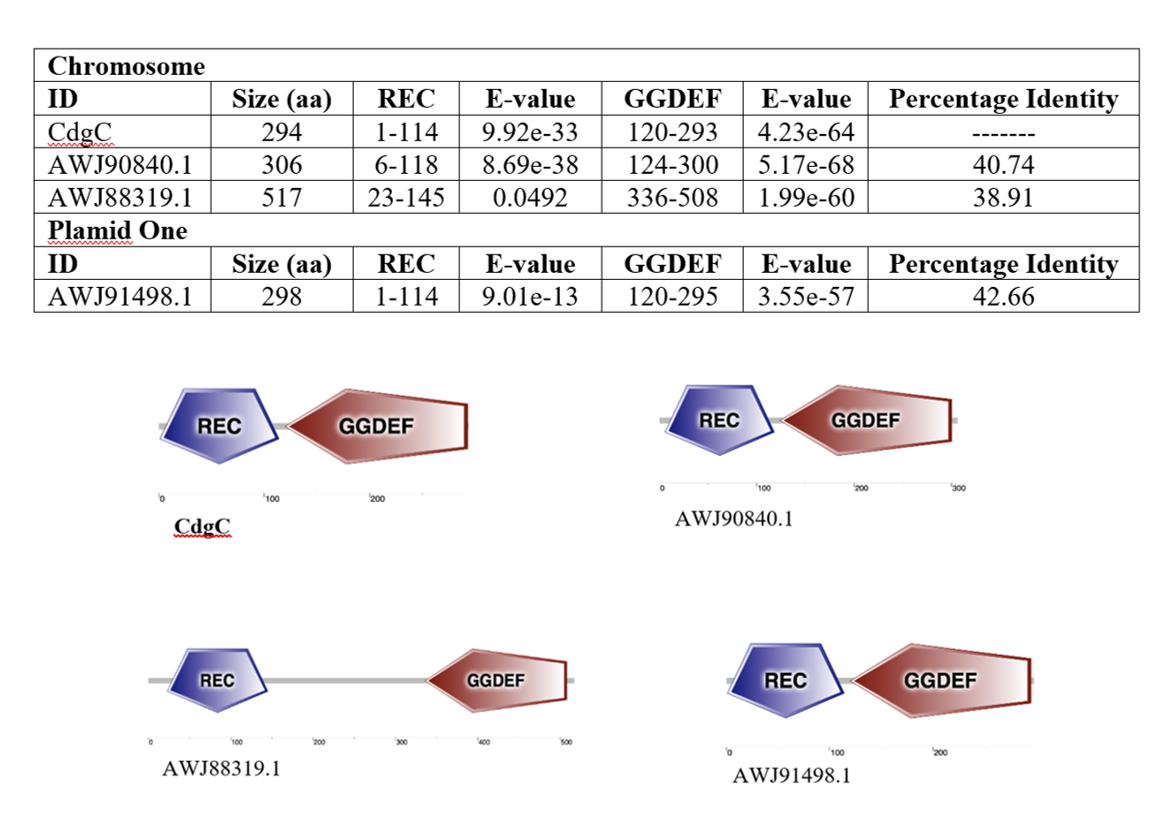
**

**Fig. S5. Redundancy of CdgC-like proteins in *A. baldaniorum*.** BLAST protein search was performed employing the amino acid sequence of CdgC as template. Domain architecture of each peptide found was performed employinh the SMART platform (5,7). Size of each sequence, the start and end position as well as the corresponding E-value of the domains by which the proteins are composed, are shown in the table. Percentage identity regarding to CdgC, is included in the last column.

**References**

1. Baldani, V. L. D., Baldani, J. I., & Döbereiner, J. (1987). Inoculation of field-grown wheat (*Triticum aestivum*) with *Azospirillum* spp. in Brazil. *Biology and Fertility of Soils*, *4*(1–2), 37–40. <https://doi.org/10.1007/BF00280348>

2. Alexandre, G., Greer, S. E., & Zhulin, I. B. (2000). Energy taxis is the dominant behavior in *Azospirillum brasilense*. *Journal of Bacteriology*, *182*(21), 6042–6048. <https://doi.org/10.1128/JB.182.21.6042-6048.2000>

3. Cruz-Pérez, J. F., Lara-Oueilhe, R., Marcos-Jiménez, C., Cuatlayotl-Olarte, R., Xiqui-Vázquez, M. L., Reyes-Carmona, S. R., Baca, B. E., & Ramírez-Mata, A. (2021). Expression and function of the cdgD gene, encoding a CHASE–PAS-DGC-EAL domain protein, in *Azospirillum brasilense*. *Scientific Reports*, *11*(1), 1–16. <https://doi.org/10.1038/s41598-020-80125-3>

4. de Castro, E., Sigrist, C. J. A., Gattiker, A., Bulliard, V., Langendijk-Genevaux, P. S., Gasteiger, E., Bairoch, A., & Hulo, N. (2006). ScanProsite: Detection of PROSITE signature matches and ProRule-associated functional and structural residues in proteins. *Nucleic Acids Research*, *34*(WEB. SERV. ISS.). <https://doi.org/10.1093/nar/gkl124>

5. Ferreira, N. D. S., Sant’ Anna, F. H., Reis, V. M., Ambrosini, A., Volpiano, C. G., Rothballer, M., Schwab, S., Baura, V. A., Balsanelli, E., Pedrosa, F. de O., Passaglia, L. M. P., de Souza, E. M., Hartmann, A., Cassan, F., & Zilli, J. E. (2020). Genome-based reclassification of *Azospirillum brasilense* Sp245 as the type strain of azospirillum baldaniorum sp. nov. *International Journal of Systematic and Evolutionary Microbiology*, *70*(12), 6203–6212. <https://doi.org/10.1099/ijsem.0.004517>

6. Kelley, L. A., Mezulis, S., Yates, C. M., Wass, M. N., & Sternberg, M. J. (2016). The Phyre2 web portal for protein modeling, prediction and analysis. *Nature Protocols*, *10*(6), 845–858. <https://doi.org/10.1038/nprot.2015-053>

7. Letunic, I., & Bork, P. (2018). 20 years of the SMART protein domain annotation resource. *Nucleic Acids Research*, *46*(D1), D493–D496. <https://doi.org/10.1093/nar/gkx922>

8. De, N., Pirruccello, M., Krasteva, P. V., Bae, N., Raghavan, R. V., & Sondermann, H. (2008). Phosphorylation-independent regulation of the diguanylate cyclase WspR. *PLoS Biology*, *6*(3), 0601–0617. <https://doi.org/10.1371/journal.pbio.0060067>

9. Sievers, F., Wilm, A., Dineen, D., Gibson, T. J., Karplus, K., Li, W., Lopez, R., McWilliam, H., Remmert, M., Söding, J., Thompson, J. D., & Higgins, D. G. (2011). Fast, scalable generation of high-quality protein multiple sequence alignments using Clustal Omega. *Molecular Systems Biology*, *7*(539). <https://doi.org/10.1038/msb.2011.75>

10. Wassmann, P., Chan, C., Paul, R., Beck, A., Heerklotz, H., Jenal, U., & Schirmer, T. (2007). Structure of BeF3--modified response regulator PleD: Implications for diguanylate cyclase activation, catalysis, and feedback inhibition. *Structure*, *15*(8), 915–927. <https://doi.org/10.1016/j.str.2007.06.016>
